# Amplification of anticancer efficacy by co-delivery of doxorubicin and lonidamine with extracellular vesicles

**DOI:** 10.1080/10717544.2021.2023697

**Published:** 2022-01-05

**Authors:** Huizhen Li, Wan Xu, Fang Li, Ru Zeng, Xiuming Zhang, Xianwu Wang, Shaojun Zhao, Jian Weng, Zhu Li, Liping Sun

**Affiliations:** a Department of Biomaterials, The Higher Educational Key Laboratory for Biomedical Engineering of Fujian Province, Research Center of Biomedical Engineering of Xiamen, College of Materials, Xiamen University, Xiamen, China; b Department of Radiotherapy, The First Affiliated Hospital of Zhengzhou University, Zhengdong Branch, Zhengzhou, China; c Key Laboratory of Marine Genetic Resources, Third Institute of Oceanography, Ministry of Natural Resources, Xiamen, China; d Department of Medical Oncology, The First Affiliated Hospital of Xiamen University, Xiamen, China; e Xiamen Nuokangde Biological Technology Co., Ltd., Xiamen, China

**Keywords:** Extracellular vesicles, doxorubicin, lonidamine, cancer therapy, lung cancer

## Abstract

Chemotherapy is commonly used for the treatment of lung cancer, but strong side effects and low treatment efficacy limit its clinical application. Here, extracellular vesicles (EVs) as natural drug delivery carriers were used to load conventional anticancer drug doxorubicin (DOX) and a chemosensitizer lonidamine (LND). Two types of EVs with different sizes (16k EVs and 120k EVs) were prepared using different centrifugation forces. We found that co-delivery of DOX and LND with both EVs enhanced the cytotoxicity and reduced the dose of the anticancer drug significantly *in vitro*. Effective delivery of anti-cancer drugs to cancer cells was achieved by direct fusion of EVs with the plasma membrane of cancer cells. On the other hand, DOX and LND inhibited cancer cell proliferation by increasing DNA damage, suppressing ATP production, and accelerating ROS generation synergistically. DOX and LND loaded EVs were also applied to the mouse lung cancer model and exhibited significant anticancer activity. *In vivo* study showed that smaller EVs exhibited higher anticancer efficiency. In conclusion, the co-delivery of the anticancer drug and the chemosensitizer with EVs may have potential clinical applications for cancer therapy.

## 1. Introduction

Lung cancer has the most commonly diagnosed cancer (11.7%) excluding female breast cancer in 2020 and was still the leading cause of cancer death (18% of the total cancer deaths in an estimated 1.8 million deaths) in the world, which threats human health severely and brings high economic burden to families (Sung et al., 2021). Chemotherapy is commonly used to treat lung cancer, but serious side effects and low therapeutic efficacy limit its application.

Doxorubicin (DOX) is an anthracycline-based broad-spectrum anticancer drug through multiple mechanisms (Figure S1(A)). It can interact with DNA in S-phase and inhibit nucleic acid synthesis. Moreover, as a DNA topoisomerase II inhibitor, DOX can cause the breakage of DNA strands. In addition, DOX is reported to produce oxygen free radicals, which causes DNA damage and lipid peroxidation (Lee et al., 2002; Swift et al., 2006). Despite these advances, severe cardiotoxicity of DOX and the development of drug resistance of cancer cells have hindered its widespread use in clinical practice (Kalyanaraman et al., 2002).

Small-molecule chemosensitizers can reverse cancer multidrug resistance (MDR) and improve the effect of chemotherapy drugs. Lonidamine (LND) is an inhibitor of hexokinaseII (HK), which is a key enzyme in the glycolysis pathway (Figure S1(B)). Cancer cells rely on aerobic glycolysis to produce ATP (Warburg effect). Thus, inhibiting the glycolysis pathway can reduce the energy supply of cancer cells and inhibit tumor growth. But the anticancer effect is limited using LND alone. Therefore, it is often used in combination with other anticancer drugs as a chemosensitizer (Floridi et al., 1981a,b). Li et al. (2002) found that the cell viability of HepG2 treated with DOX and LND for 24 h was reduced by 40% compared with doxorubicin alone. However, due to their small molecular weight and size, they are rapidly cleared by the body. Moreover, poor solubility and low permeability limited their accumulation in cancer cells (Sun et al., 2017).

To solve these problems, different carriers are used to deliver chemotherapeutic drugs to improve the efficacy of cancer treatment. Conventional delivery systems include liposomes (Yu et al., 2018), dendrimers (Cruz et al., 2020), inorganic nanomaterials (Sun et al., 2015), micelles (Nasongkla et al., 2006), virus-like protein particles (Zhao et al., 2020), etc. Although these synthetic carriers enhance anticancer efficacy and reduce drug resistance, they have also some disadvantages, which limit their applications. The main disadvantages of conventional nanocarriers are their toxicity and low biocompatibility. Artificial nanoparticles have non-uniform particle size and are prone to form agglomerates which can be rapidly cleared from the blood circulation by the mononuclear phagocyte system. Some nanomaterials or their metabolites show toxicity and potential side effects on human health. Moreover, complicated synthesis and purification process, lack of targeting, induction of immune response, and slow metabolism in the body also hinder their clinical applications (Ping & Mumper, 2013; Garofalo et al., 2018; Cabeza et al., 2020; Huyan et al., 2020; Witwer & Wolfram, 2021). Recently, extracellular vesicles (EVs) have been used as natural drug delivery carriers. Compare with artificial liposomes and nanomaterials, EVs demonstrate high biocompatibility, low immunogenicity, and high delivery efficiency (Chen et al., 2019). EVs are cell-derived nano-scale vesicles mediating cell-cell communication. These vesicles can be passively transported to the tumor site through enhanced permeability and retention (EPR) effect. EVs carrying siRNA specific to oncogenic KRAS mutation have suppressed pancreatic cancer, inhibited metastasis, and increased overall survival (Ha et al., 2016; Kamerkar et al., 2017). In addition, EVs loaded with DOX (DOX-EVs) showed no cardiotoxicity as observed in DOX-treated mice (Toffoli et al., 2015).

Although DOX-EVs are safer than free DOX, its efficacy for cancer treatment still needs to be improved. In this study, we used EVs to load DOX and LND simultaneously. Co-delivery of DOX and LND with EVs (DOX/LND-EVs) enhanced the cytotoxicity and further reduced the dose of the anticancer drug. The mechanism of increased anti-cancer effect was explored. Effective delivery of anti-cancer drugs to cancer cells was achieved by direct fusion of EVs with the plasma membrane of cancer cells. On the other hand, DOX and LND inhibited cancer cell proliferation by increasing DNA damage, suppressing ATP production, and accelerating reactive oxygen species (ROS) generation synergistically. DOX and LND loaded EVs were also applied to the mouse lung cancer model and significantly suppressed *in vivo* tumor growth and reduced the toxicity. Co-delivery of anticancer drugs and chemosensitizer with EVs may have potential clinical application for cancer therapy.

## 2. Materials and methods

### 2.1. Materials and reagents

LND was purchased from Macklin (Shanghai, China). Doxorubicin hydrochloride (DOX), Methylthiazolyldiphenyl-tetrazolium bromide (MTT), Dimethyl sulfoxide (DMSO) were obtained from Sangon Biotechnology (Shanghai, China). Typan Blue Staining Cell Viability Assay Kit, DNA ladder extraction kit, ATP Assay Kit, Reactive Oxygen Species Assay Kit, BCA protein assay kit, 3,3′-dioctadecyloxacarbocyanine perchlorate (DiO) was purchased from Beyotime (Shanghai, China). The RPMI 1640 medium and penicillin-streptomycin were purchased from BBI (Canada). The fetal bovine serum (FBS) was purchased from ExCell Biological Technology (Taicang, China). The 0.22-μm pore-size syringe filters were from GVS Filter Technology (USA).

### 2.2. Cell culture

Non-small cell lung carcinoma (NSCLC) A549 cells were obtained from the American Type Culture Collection (ATCC). Exosome-depleted FBS was prepared as follows: 10% fetal bovine serum (FBS) was centrifuged at 100,000 ×g for 70 min to remove bovine EVs, followed by filtration through the 0.22 μm syringe filter (Pi et al., 2018). A549 cells were cultured in 150-mm diameter petri dishes at 37 °C in a 5% CO_2_ incubator using RPMI 1640 medium supplemented with exosome-depleted FBS and 1% penicillin–streptomycin (100 units/mL penicillin and 100 μg/mL streptomycin).

### 2.3. Isolation of EVs

When the cells reached a confluency of ∼90% (after ∼48 h), the conditioned cell culture medium was collected, centrifuged for 10 min at 300 g to get rid of dead cells, and then centrifuged for 30 min at 3000 g to remove cell debris. Subsequently, the supernatant was further centrifuged for 45 min at 16,000 g to pellet 16k EVs (Guo et al., 2019). At last, the collected culture medium was subjected to ultracentrifugation at 4 °C 120,000 g for 90 min to isolate 120k EVs using Beckman Coulter XE-90K (Beckman, USA). All the pellets were resuspended in phosphate-buffered saline (PBS) and then submitted to the second ultracentrifugation in the same conditions. Afterward, the supernatant was discarded and the final pellet was resuspended in 50–100 μL PBS for the following experiments. Exosome protein content was quantified using the bicinchoninic acid (BCA) method.

### 2.4. Characterization of EVs

EVs were negatively stained and observed using a Hitachi HT-7800 transmission electron microscope (TEM) at 200 kV. The concentration and size distribution of purified EVs were measured by a laboratory-built nano-flow cytometer. Expression of EVs markers (CD63, TSG101) was evaluated in EVs derived from A549 cells by western blotting.

### 2.5. Preparation and characterization of drug-loaded EVs

Purified EVs from 80 mL cell culture medium were mixed with DOX, LND, or DOX/LND and incubated overnight at 4 °C, then centrifuged at 16,000 g for 1 h or ultracentrifuged at 120,000 g for 90 min at 4 °C to remove excess drugs unwrapped into EVs. The pellets were collected and resuspended in PBS. DOX-EVs were observed under the fluorescent microscope. The content of DOX and LND in EVs was quantified using a multifunctional microplate reader (Infnite-M200 PRO, TECAN). The intrinsic fluorescence of DOX was measured at 594 nm with excitation at 494 nm. The ultraviolet absorbance of LND was analyzed at 299 nm. Drug-loaded EVs were negatively stained and observed using TEM at 200 kV. Zeta potentials were measured with a Mastersizer 2000 (Malvern, UK). The encapsulation efficiency was calculated using the following formula:

$$\text{EE}\;(\%)\;=\;\frac{\text{mass}\;\text{of}\;\text{drug}\;\text{in}\;\text{EVs}}{\text{mass}\;\text{of}\;\text{drug}\;\text{added}}\;\times 100\%$$

### 2.6. *In vitro* cytotoxicity assay

To explore the cytotoxicity, A549 cells were treated with DOX, DOX/LND, DOX-EVs, or DOX/LND-EVs. MTT assays were performed. Firstly, A549 cells were seeded in a 96-well plate at a density of 5000 cells per well. When the cells reached 80–90% confluency, the medium was removed and cells were washed twice with PBS. Next, a fresh medium containing various concentrations of DOX, LND, or drug-loaded EVs was added and incubated for 36 h. The supernatant was discarded and the cells were washed with PBS to remove the residual drug. 200 µL of 0.5 mg/mL MTT was added and further incubated for 4 h. Finally, MTT in the wells was completely removed and replaced with 200 µL of DMSO. The microplate was shaken on a shaker at 37 °C for 20 min. The viability of the cells was determined by measuring the absorbance of each well at 490 nm using a multi-functional microplate reader. The cell viability was determined by comparing the mean absorbance of treated cells to untreated cells. A graph was plotted with percentage viability *vs.* concentration of the tested compound and the half-inhibitory concentration (IC_50_) values were calculated by SPSS 25.0.

### 2.7. Cell uptake and localization

To investigate the cellular uptake efficiency of various samples, A549 cells were treated with free DOX, DOX-EVs (equivalent to 1 µg/mL DOX) for different times (1, 6, 12, 24, and 36 h). Untreated cells were used as control. After incubation, about 1.8 × 10^5^ cells were trypsinized, resuspended in PBS, then the fluorescence signals in cells were analyzed using flow cytometry (Coulter EPICS XL, Beckman coulter). To probe the internalization mechanism of EVs, EVs were labeled with DiO green membrane fluorescent dye. A549 cells were seeded into 24-well plates at a density of 10^4^ cells/well and cultured for 24 h, then the cells were incubated with free DOX, DiO-labeled EVs (DiO-EVs), DOX-EVs, DiO-labeled DOX/EVs (DiO/DOX-EVs) (equivalent to 1 µg/mL DOX) for 36 h. After 36 h, the medium was removed and cells were washed three times with PBS and fixed with 4% paraformaldehyde for 30 min. The images were recorded using a confocal laser-scanning fluorescence microscope (TCS P5, Leica). The uptake efficiency of DOX and LND by the cells was measured by a multi-functional microplate reader. Firstly, DOX/LND, DOX/LND-EVs were incubated with A549 cells for 36 h. After washing with PBS, 200 µL of cell lysate was added to each well. The cells were lysed and centrifuged at 12,000 g 4 °C for 5 min. Then the supernatant was collected for quantitative analysis. The fluorescence of DOX was measured at 594 (excitation 494 nm). The absorbance of LND was analyzed at 299 nm.

### 2.8. The mechanisms of synergistic anticancer effect

To confirm the extent of DNA damage, a DNA ladder experiment was performed. Briefly, A549 cells were seeded into 6-well plates, and the medium was discarded when cells reached 100% of the entire wells. Then DOX, DOX/LND, DOX-EVs, DOX/LND-EVs were added and incubated with cells for 36 h. After incubation, cells were digested with trypsin, washed with PBS, collected by centrifuging at 3000 g for 2 min. Finally, DNA in the cells was extracted using a DNA Ladder extraction kit. DNA fragmentation was visualized by 1% agarose gel electrophoreses stained with GelRed.

The change of ATP content in the cancer cells was measured by an ATP Assay Kit. Briefly, cells were seeded into 24-well plates and incubated for 24 h. Then LND, DOX/LND, LND-EVs, or DOX/LND-EVs were added and incubated for 36 h, respectively. Forty microliters of lysate was added to each well to lyse cells at 4 °C. After centrifugation at 12000 g for 5 min, 10 µL of supernatant was collected to determine the ATP content of each group according to the instructions of the Kit.

The reactive oxygen species (ROS) in the cancer cells was measured by a ROS detection kit. Firstly, cells were seeded into 12-well plates at 2 × 10^4^ cells per well and incubated for 24 h. Then the cells were incubated with DOX, DOX/LND, DOX-EVs, and DOX/LND-EVs for 36 h, respectively. The supernatant was discarded and 800 µL of 10 µM DCFH-DA probe solution was added to each well and incubated for 30 min at 37 °C. The cells were washed to remove the probes that have not entered the cells. Then the cells were digested from the wells, centrifuged at 3000 rpm for 2 min. Finally, the cell pellet was resuspended with a 200 µL 1640 culture medium, and the fluorescence intensity of each group was measured at the excitation wavelength of 488 nm and the emission wavelength of 525 nm by a microplate reader.

### 2.9. *In vivo* animal trials

Six to eight-week-old female BALB/c nude mice were purchased from Beijing Vital River Laboratories (*Beijing*, China). Subcutaneous lung cancer mouse models were established by subcutaneous injection of A549 cells (5 × 10^6^ cells in 200 µL PBS per mouse) into the right leg flank. When the tumor volume reached an average size of about 80 mm^3^ (volume = length × width^2^ × 0.5) (Li et al., 2016; Yong et al., 2019), the mice were randomly sorted into different experimental groups (*n* = 5 per group) and intravenously injected with PBS (control), DOX, DOX/LND, DOX-16k EVs, DOX-120k EVs, DOX/LND-16k EVs, DOX/LND-120k EVs (equivalent to 0.1 mg/kg DOX), and high dosage DOX (4 mg/kg) every three days for a total of 5 injections. Tumor volumes were measured every 2 days *via* vernier caliper and the weights of mice were also recorded. Relative tumor volumes (RTV) were calculated according to the following formula: RTV = *V_n_*/*V*_0_, where *V_n_* and *V*_0_ are the tumor volume at day n and day 0, respectively. On day 16, blood was collected from the eyes and centrifuged at 3000 rpm for 5 min to obtain the serum. The aspartate aminotransferase (AST) and creatine kinase MB isoenzyme (CK-MB) were analyzed by Wuhan Servicebio Technology Co., Ltd. (Xiamen, China). Then the mice were sacrificed, tumors and major organs (heart, liver, spleen, lung, and kidney) were washed with PBS and weighed. The tissues were fixed with 4% paraformaldehyde, paraffin-embedded, sectioned, stained with hematoxylin and eosin (H&E), and observed under a microscope.

## 3. Results and discussions

### 3.1. Preparation and characterization of EVs and drug-loaded EVs

**Figure 1.**
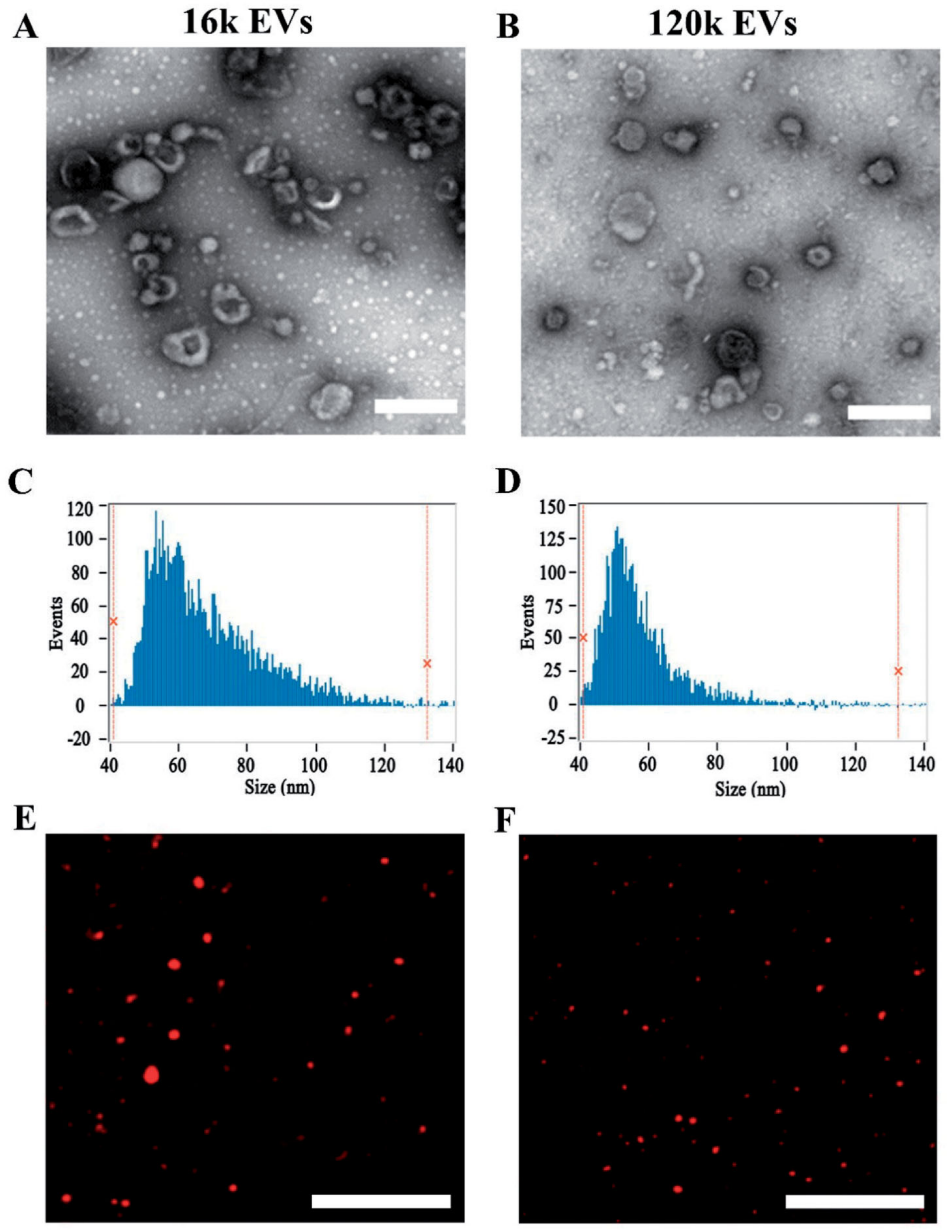
Characterization of EVs and drug-loaded EVs. TEM image of 16k EVs (A) and 120k EVs (B). Scale bar: 200 nm. Size distribution of 16k EVs (C) and 120k EVs (D) measured by nano-flow cytometer. Fluorescence image of 16k EVs (E) and 120k EVs (F). Scale bar: 20 µm.

In this study, EVs were isolated by differential centrifugation. Two populations of EVs (16k EVs and 120k EVs) were prepared under different centrifugation forces. TEM images showed irregular round or oval shapes of both EVs (Figures 1(A,B)). The diameters of 16k EVs and 120k EVs were mostly distributed in the range of 50–200 nm. The mean size of 16k EVs obtained by high-speed centrifugation was 67.96 ± 16.27 nm (Figure 1(C)). The diameters were distributed in the range of 40–120 nm. While the 120k EVs obtained by ultracentrifugation exhibited smaller size (57.18 ± 10.91 nm) nm (Figure 1(D)). To assess the surface marker TSG101 (tumor susceptibility gene 101 protein) and tetraspanins CD63, we performed Western blots of A549 cell lysate, 16k EVs and 120k EVs. Both markers were rich in 120k EVs and cells, but poor in 16k EVs (Figure S2), indicating the different origin of the EV populations. The small subtype 120k EVs bearing TSG101 and CD63 could be exosomes mainly originated from multivesicular bodies (MVBs), while the bigger 16k EVs might be microvesicles originated directly from the plasma membrane (Mathieu et al., 2019).

DOX-loaded EVs were observed under a fluorescence microscope. Figure 1(F) showed that DOX-EVs emitted strong red fluorescence of DOX, confirming encapsulation of DOX in both types of EVs. In addition, the amount of DOX and LND loaded in the DOX/LND-EVs gradually increased with the initial dosage of drugs co-incubated with EVs (Figures S3(A,B)). TEM images showed that the morphology of EVs remained unchanged after drug loading (Figure S4). Nano-flow cytometry showed a slight increase in size. The DOX/LND-16k EVs were in the mean size of 93.2 ± 24.2, while the DOX/LND-120k EVs were 70 ± 11.1 nm (Figures S5(A,B)). The zeta potentials of both vesicles were negative due to negatively charged phospholipid of the membrane (Figure S6) since the major lipid species of EVs include glycosphingolipids, sphingomyelin, cholesterol, and phosphatidylserine, which are either neutral or negatively charged (Skotland et al., 2019). The zeta potential of the 16k EVs and 120k EVs were −15.2 and −15.9 mV, respectively. No obvious changes were observed after drug loading because DOX and LND were encapsulated into the vesicles and excess drugs were washed away by ultracentrifugation. The encapsulation efficiencies of DOX and LND in 16k EVs were 0.81 ± 0.22 and 4.16 ± 1.9%, while those of 120k EVs were 0.43 ± 0.03 and 2.77 ± 0.35%, respectively. In the future, new loading strategies, such as sonication and extrusion-assisted active loading could be used to achieve more efficient loading (Chen et al., 2021). To test the stability of EVs, we measured the absorbance of released DOX and LND from EVs and found little change of the absorbance in 22 h, indicating EVs were stable to prevent the drugs from leakage (Figure S7). These results indicated that EVs extracted from A549 cells could be used as DOX and LND carriers.

### 3.2. *In vitro* anticancer efficacy

**Figure 2.**
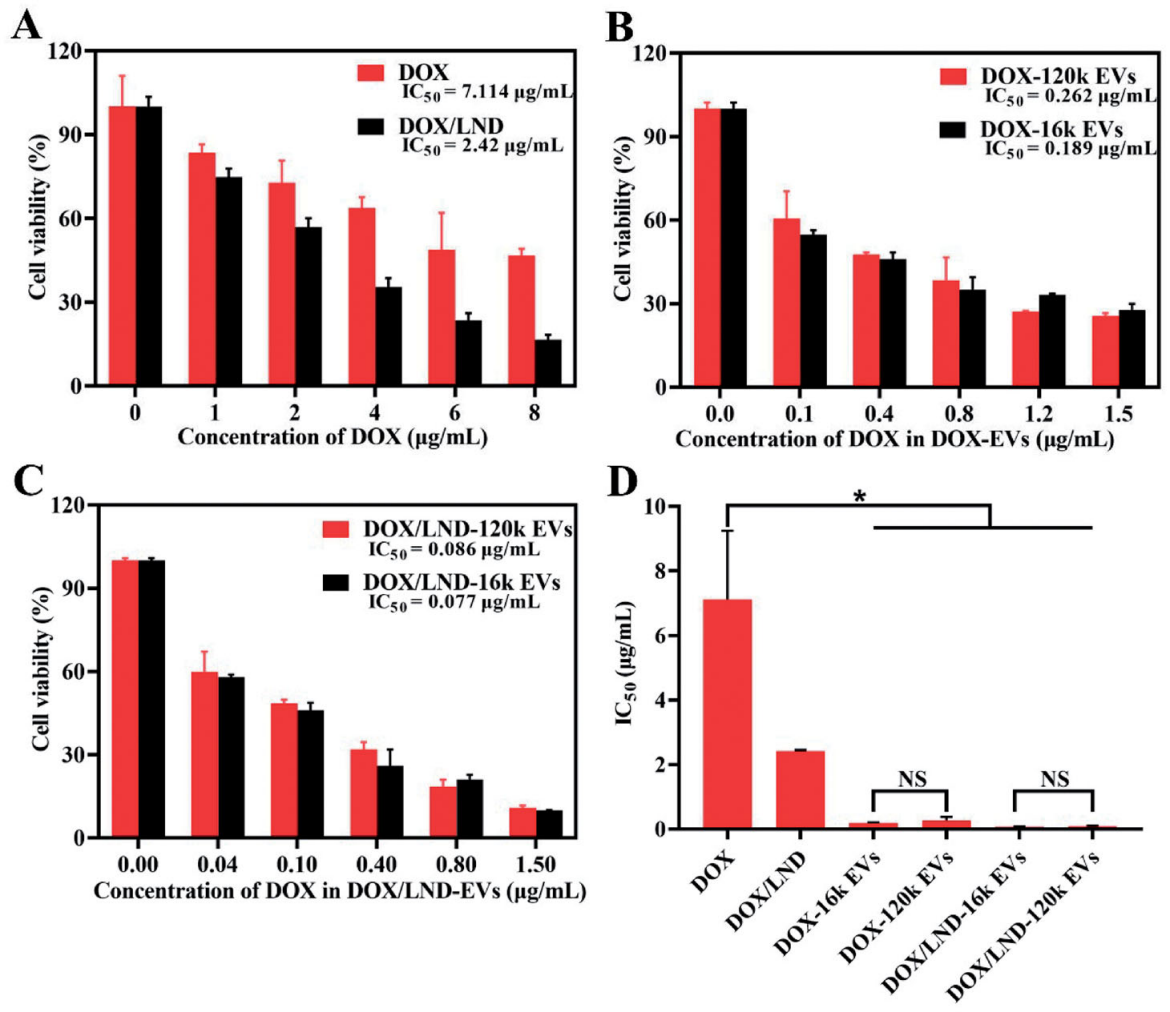
Anticancer efficacy of free DOX and DOX/LND (A), DOX-EVs (B), and DOX/LND-EVs (C). (D) Comparison of IC_50_ of different groups. Data were analyzed with parametric *t*-test and presented as mean ± *SD*, *n* = 3 (*p < .05, **p < .01, NS: non-significant).

To explore whether the combined delivery of DOX and LND with extracellular vesicles could enhance the anticancer effect, we investigated the cell viability of A549 treated with DOX, DOX/LND, or DOX/LND-EVs. The mass ratio of DOX/LND (1:6) was used as an optimized ratio according to the results of the MTT test (Figure S8). With the increase of DOX concentration, the cell activity gradually decreased. Treatment with DOX alone showed IC_50_ (the concentration necessary to produce 50% inhibition of cell growth) value of 7.114 µg/mL (Figure 2(A)). On the one hand, the combination of the anticancer agent DOX with the chemosensitizer LND increased the cytotoxic effect (IC_50_ 2.42 µg/ml); on the other hand, loading of DOX in EVs also enhanced the inhibition of the A549 cell proliferation obviously. The IC_50_ values of DOX-16k EVs and DOX-120k EVs decreased to 0.189 and 0.262 µg/mL, respectively, which were significantly lower than that of DOX (Figure 2(B)). Finally, encapsulation of both DOX and LND in EVs further inhibited cancer cell proliferation (Figure 2(C)). The IC_50_ values of DOX/LND-16k EVs and DOX/LND-120k EVs tend to be 92- and 83-fold lower than IC_50_ of DOX (Table S1), indicating that co-delivery of DOX and LND with extracellular vesicles could significantly reduce the dose of chemotherapy and increase the anti-cancer effect, thus reducing the side effects. The cytotoxic effects showed the following order: DOX < DOX/LND < DOX-EVs < DOX/LND-EVs. *In vitro* study didn’t exhibit a significant difference of IC_50_ between the big vesicles (16k EVs) and small vesicles (120k EVs) (Figure 2(D)). In conclusion, loading of DOX and LND into both big and small EVs could effectively amplify the anticancer efficacy *in vitro* (Figure S9).

### 3.3. Cellular uptake of EVs

**Figure 3.**
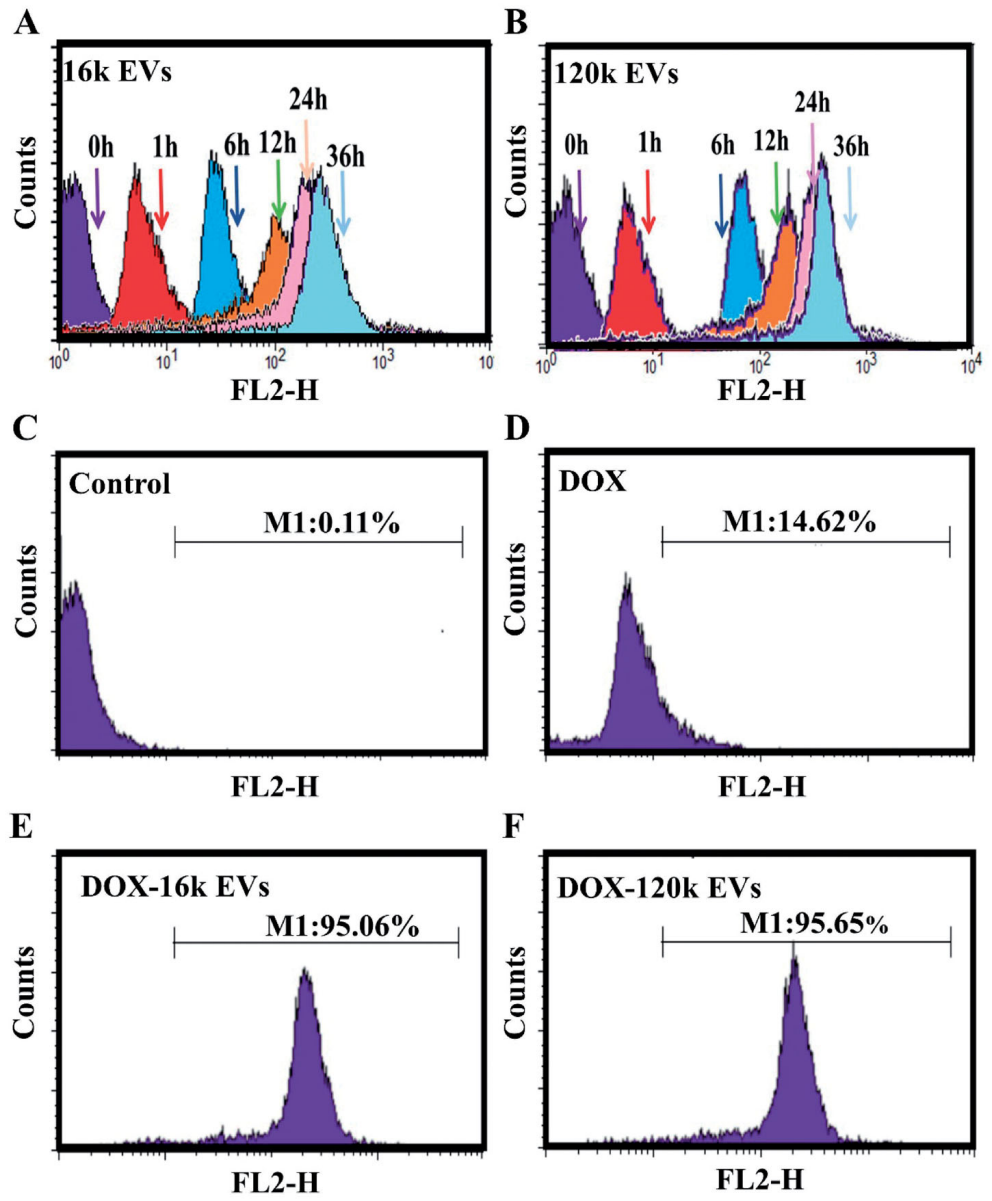
Flow cytometric evaluation of the cellular uptake of DOX-16K EVs (A) and DOX-120K EVs (B) at 0, 1, 6, 12, 24, and 36 h. The cellular uptake of the control group (C), free DOX (D), DOX-16k EVs (E), and DOX-120k EVs (F) at 36 h. The concentration of DOX is 1 µg/mL.

Based on the red auto-fluorescence of DOX, flow cytometry was used to quantify the uptake of free DOX or DOX-EVs by the cells. A549 cells treated with DOX-EVs showed that both EVs were internalized by the cell in a time-dependent manner (Figures 3(A,B), Figure S10). Fluorescent signals were detected at 1 h, indicating early uptake of DOX-EVs. At 36 h, the cellular uptake of DOX-EVs reached saturation. Therefore, in the following experiments, DOX-loaded EVs were incubated with cells for 36 h. Untreated cells showed little fluorescence (Figure 3(C)). The uptake rates of DOX-16k EVs (95.06%) and DOX-120k EVs (95.65%) were significantly higher than that of DOX alone at the same concentration (14.62%) (Figures 3(D–F)), indicating that the cellular uptake of DOX encapsulated by EVs was more efficient than that of free drug. This result was consistent with the cytotoxicity experiments.

In addition, we quantitatively analyzed the cellular uptake of drugs using a microplate reader. The concentrations of DOX taken up by A549 cells in the DOX/LND, DOX/LND-16k EVs and DOX/LND-120k EVs group were 0.11, 0.84, and 0.79 µg/mL, respectively. Meanwhile, the cellular uptakes of LND of the three groups were 2.57, 4.45, and 3.61 µg/mL, respectively (Table S2), confirming that EVs enhanced cellular uptake of drugs. The efficiency of DOX taken up by the cells was significantly lower than LND for the DOX/LND group (11 *vs.* 44%). This difference might be attributed to their physicochemical properties. The doxorubicin hydrochloride (DOX) we used is water-soluble and enters the cell by simple diffusion (Xu et al., 2014), while LND is more easily to enter cells due to its hydrophobic property and preferential interaction with lipid membranes (De Martino et al., 1987). However, when encapsulated in extracellular vesicles, the efficiency of DOX and LND taken up by the cells increased significantly (from 11 to 84 and 79% for 16k EVs and 120k EVs, respectively), indicating that EVs as drug carriers could improve the uptake efficiency of both drugs, especially the water-soluble DOX. These results confirmed the high efficiency of drug delivery by EVs.

### 3.4. The pathway of extracellular vesicles into cells

**Figure 4.**
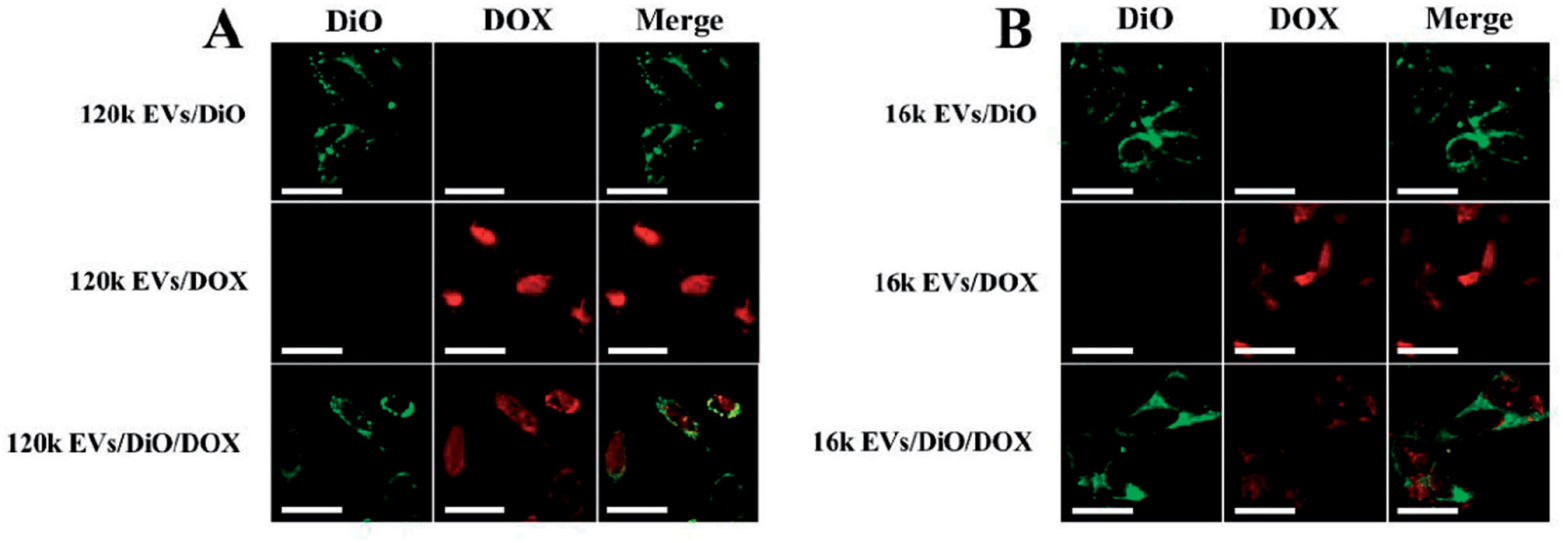
Cell internalization assay of 120k EVs (A) and 16k EVs (B) using confocal microscopy (Scale bar: 20 μm).

We investigated the internalization mechanism of EVs. EVs cargoes can enter cells either directly via fusion with the plasma membrane (Van den Boorn et al., 2011; Zheng et al., 2019), or by endocytosis (Mulcahy et al., 2014; Li et al., 2018). To explore the exact internalization mechanisms in our study, A549 cells were incubated either with DiO-labeled EVs (a green fluorescent lipid membrane dye), or DOX-loaded EVs, or DiO and DOX double-labeled EVs. When EVs internalization occurs via the membrane fusion pathway, the loaded DOX is released into the cell, while the fluorescent dye DiO remains on the cell membrane surface, resulting in green fluorescence on the cell membrane and red fluorescence emitted by DOX in the cell. However, if EVs internalization occurs via endocytosis, EVs enter the cytoplasm and only show red fluorescence in the cell. We observed that red fluorescence emitted by DOX was mainly distributed inside the cells, while green fluorescence was localized on the cell membrane rather than in the cytoplasm (Figure 4). These results imply that EVs may enter the cell through direct membrane fusion since the fluorescent dye on the extracellular vesicle membrane was retained on the cell membrane after being uptaken by the A549 cells. Our results are consistent with previous studies (Prada & Meldolesi, 2016; Zheng et al., 2019).

### 3.5. The mechanisms of synergistic anticancer effects

#### 3.5.1. Inhibition of intracellular DNA synthesis

**Figure 5.**
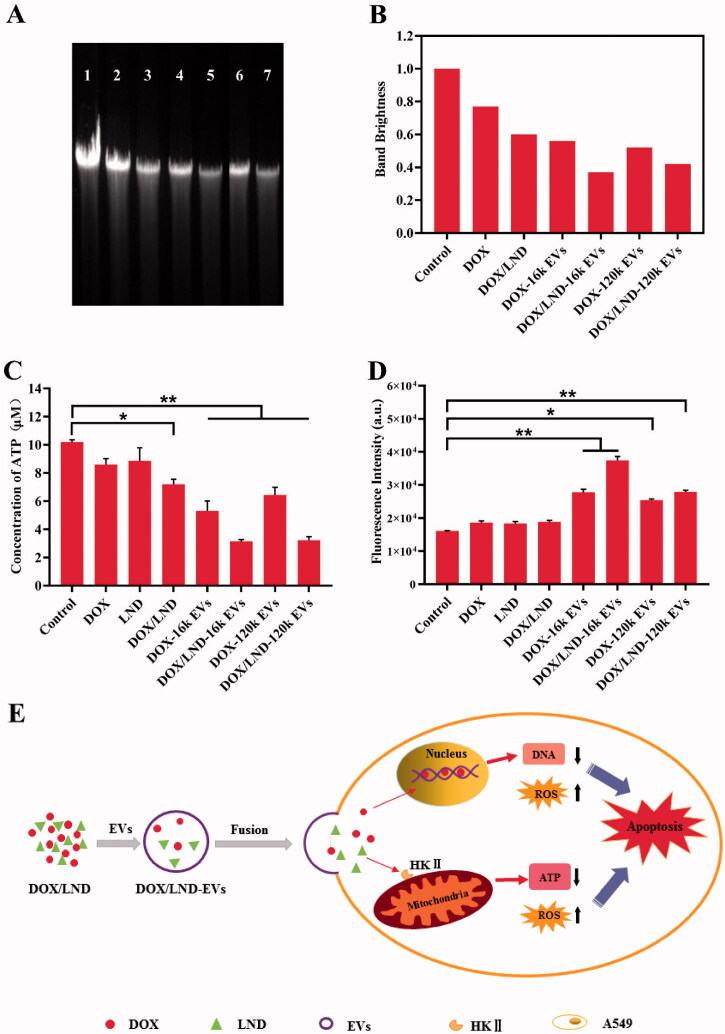
Synergistic anticancer effects of DOX/LND-EVs. (A) Electrophoresis of DNA of A549 cells treated with (1) Negative control without treatment; (2) DOX; (3) DOX/LND; (4) DOX-16k EVs; (5) DOX-120k EVs; (6) DOX/LND-16k EVs; (7) DOX//LND-120k EVs. (B) Quantitative analysis of the brightness of each band in (A). The brightness of band 1 is defined as 1. (C) Chemiluminescence assay of intracellular ATP. *p < .05, **p < .01. (D) Fluorescence assay of ROS. Data were analyzed with parametric *t*-test and presented as mean ± *SD*, *n* = 3 (*p < .05, **p < .01). (E) The scheme of potential mechanisms of co-delivery of DOX and LND by extracellular vesicles to enhance the anticancer effect.

DOX affect DNA transcription by forming a complex with DNA, which obviously inhibits the synthesis of nucleic acids. On the other hand, it can also inhibit topoisomerase II, resulting in the break of DNA double strands (Swift et al., 2006). Therefore, we compared the extents of DNA damage of different groups. As shown in Figures 5(A,B), the brightness of the control group without treatment (band 1) was significantly higher than other experimental groups treated with different combinations of drug and EVs, while DOX/LND-EVs exhibited the weakest bands (band 5 and 7). The extents of DNA damage followed the same order as the results of cytotoxicity assay: DOX < DOX/LND < DOX-EVs < DOX/LND-EVs. These results indicated that loading chemotherapy drugs in extracellular vesicles were more effective in inhibiting DNA synthesis compare with free drugs.

#### 3.5.2. Inhibition of intracellular ATP

We examined the effects of different groups on cellular ATP levels using a chemiluminescence detection kit. There was no significant difference between LND and the control group, indicating that LND alone had little effect on ATP synthesis. But when LND was combined with DOX, the intracellular ATP synthesis was significantly reduced (*p* < .05) (Figure 5(C)). DOX-loaded EVs also showed reduced intracellular ATP concentration. Furthermore, co-delivery of LND and DOX with extracellular vesicles had the most effective inhibition of ATP synthesis (*p* < .01). These results confirmed that the combination of LND and DOX in EVs significantly inhibited the energy metabolism of cancer cells through a synergistic effect.

#### 3.5.3. Impact of intracellular ROS

ROS, including singlet oxygen (^1^O_2_), superoxide anion (•O^2−^), hydroxyl radical (•OH), and hydrogen peroxide (H_2_O_2_), are highly reactive molecules mainly released from mitochondria. A variety of chemotherapy drugs achieve cancer suppression through ROS-dependent apoptosis (Juan et al., 2008). In this study, we used a fluorescent dye 2′,7′-dichlorofluorescin diacetate (DCFH-DA) to detect ROS in cancer cells. All groups with EVs carriers showed higher fluorescence intensity than the control group (Figure 5(D)). The fluorescence intensity of DOX-16k EVs and DOX-120k EVs was significantly higher than that of DOX (*p* < .01). Meanwhile, the fluorescence intensity of DOX/LND-16k EVs and DOX/LND-120k EVs were significantly increased compared with DOX/LND (*p* < .01). These results confirmed that the loading of drugs in EVs could enhance the cellular ROS level due to more effective delivery of drugs by EVs.

Despite the improvements made in the last years in chemotherapy of tumors, severe side effects and MDR remain a problem. Our system represents a strategy for efficient delivery of the anticancer drug DOX and chemosensitizer LND by the cancer cells originated EVs, which enhances the efficacy of chemotherapy significantly and forms a potentially powerful anticancer tool. The potential mechanisms of synergistic anticancer effect of DOX/LND-EVs were summarized in Figure 5(E). Firstly, DOX and LND are encapsulated in EVs and delivered to human lung cancer cells through membrane fusion between EVs and cells. DOX enters the nucleus and mitochondria, inhibiting DNA synthesis (Lee et al., 2002). Meanwhile, LND inhibits HKII in the glycolysis pathway, thereby reducing the ATP supply of the cancer cells (Floridi et al., 1981b). In addition, previous studies suggest that DOX and LND induced cell death due to the generation of ROS, which could cause oxidative damage of mitochondrial proteins, cell membranes, and DNA (Eom et al., 2005; Guo et al., 2016). In conclusion, DOX, LND, and EVs synergistically promoted cancer cell apoptosis.

The other mechanisms mediating the synergistic anticancer effect of LND and DOX include the following: (1) LND induced intracellular acidification so that free basic DOX (pKa ∼8.5) would be more readily captured in the acidified tumor cells; (2) LND-induced ATP deprivation decreases the supply of energy for MDR efflux pumps, resulting in more drugs retained in tumor cells; (3) mitochondria-dependent apoptosis; (4) antiangiogenic effect; (5) effective killing of cancer stem cells. These multiple mechanisms functioned together to kill tumor cells efficiently (Floridi et al., 1998; Huang et al., 2020).

It has been reported that encapsulation of DOX and LND in modified redox-sensitive liposomes presented a synergistic effect on glioma. *In vitro* tests showed a remarkable interference to mitochondria, such as reducing intracellular ATP production and inducing ROS generation (Peng et al., 2021). Additionally, it has been demonstrated that LND/DOX combined with mitochondria-targeting copolymer triphenylphosphine (TPP) showed a synergistic anticancer effect. TPP-LND-DOX nanoparticles could induce significant ROS production, mitochondrial membrane potential decrease, mitochondrial apoptosis pathway, and conquered MDR in cancer therapy (Liu et al., 2017). These results are in accordance with our study.

### 3.6. *In vivo* anticancer efficacy

**Figure 6.**
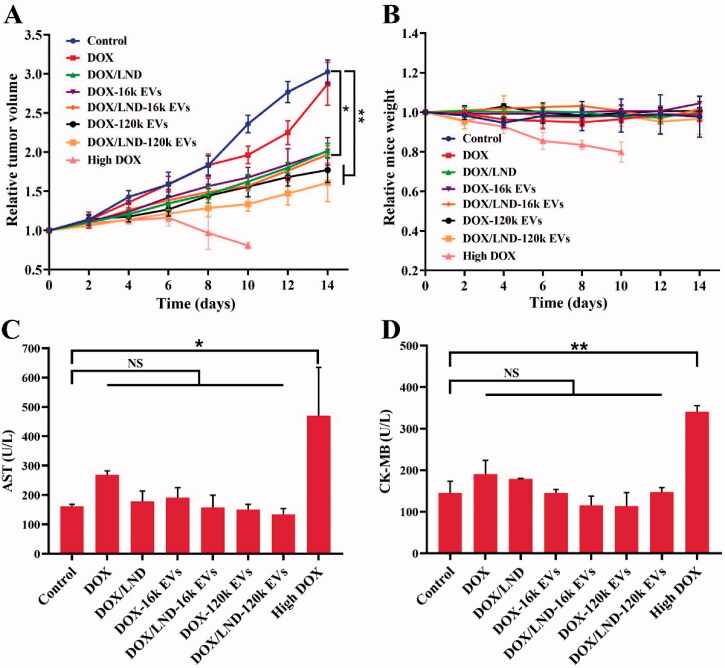
*In vivo* antitumor efficacy of DOX/LND-EVs in BALB/c nude mice. (A) Relative tumor volume over time. (B) Relative mice weight over time. (C) Serum AST. (D) Serum CK-MB. Data were analyzed with non-parametric test and presented as mean ± *SD*, *n* = 5 (*p < .05, **p < .01, NS: non-significant).

A549 EVs were used as drug delivery tools for anti-lung cancer therapy because cancer-derived EVs showed a selective tropism for the tumor tissue from which the vesicles originated (Toffoli et al., 2015; Yong et al., 2019; Gaurav et al., 2021). The *in vivo* anticancer effects were studied with nude mice bearing human lung cancer. Compared with DOX alone, the combination of DOX and LND reduced the tumor volume significantly (*p* < .05). More significant tumor suppression was observed when DOX and LND were loaded in EVs (*p* < .01), demonstrating synergistic anti-tumor effects achieved by co-delivery of DOX and LND with EVs (Figure 6(A), Table S3). A similar tendency was observed in the size of those harvested tumor tissues and tumor weight of tumor-bearing mice (Figures S11(A,B)). Based on the body weights of the mouse groups over time (Figure 6(B)), the doses of 0.1 mg/kg DOX formulations were well tolerated and the DOX-based carriers did not show significant toxicity to the test mice. The serum AST and CK-MB, biomarkers of myocardial injury, displayed no significant difference compared with the control group (Figures 6(C,D)). H&E staining also showed no obvious tissue damage. These results suggest that EVs are safe and effective drug delivery carriers for lung cancer treatment. In comparison, although high DOX exhibited a significant tumor growth inhibition, the mice showed severe weight loss and died on the tenth day (Figure 6(B)). Moreover, AST and CK-MB increased significantly. Histopathological results showed swelled and disordered myocardial fiber (Figure S11(C)). The above results indicated that high dosage DOX caused severe cardiac damage.

The better anticancer efficiency of EVs-mediated drug delivery might be attributed to the protection of drugs by EVs and their specific tumor-targeting ability (Gaurav et al., 2021). The autologous exosomes can reach the mother cells more specifically and play a targeted role. Moreover, EVs can easily extravasate from the vascular leak of tumor vessels due to enhanced permeability and retention effect (Hoshino et al., 2015; Yong et al., 2020). Wang et al. reported that docetaxel-loaded exosomes derived from A549 cells exhibited excellent tumor targeting ability. *In vivo* fluorescence imaging results revealed that drug-loaded EVs reached the tumor site and suppressed tumor development in the A549 mouse model (Wang et al., 2021). The above studies confirmed the tumor-targeting ability of EVs.

Among all groups, DOX/LND-120k EVs exhibited the most significant anticancer activity, with 53 and 47% of relative tumor volume (RTV) and tumor weight (RTW) (Table S3). DOX/LND-120k EVs were more efficient than DOX/LND-16k EVs (RTV 65%, RTW 70%), which is different from the results of *in vitro* cytotoxicity assay. Owing to the limitations of the isolation method of EVs (differential centrifugation), 16k EVs isolated by low-speed centrifugation could be heterogeneous vesicles mainly containing bigger microvesicles originating from the plasma membrane. While 120k EVs isolated through ultra-high-speed centrifugation composed mostly of small EVs generated by multivesicular bodies, which was confirmed by their surface markers TSG101 and CD63 (Figure S2). 120k EVs from MVBs could escape uptake by the reticuloendothelial system (RES) and therefore, efficiently target tumor cells, resulting in higher tumor accumulation and penetration (Gao et al., 2021). There is no RES in the cell culture system, thus 120k EVs and 16k EVs-loaded drugs showed no difference in the *in vitro* anticancer activity.

## 4. Conclusions

Herein, we obtained 16k EVs and 120k EVs secreted by A549 cells with an average size of 68 and 53 nm, respectively. Co-delivery of chemotherapeutic DOX and chemosensitizer LND with EVs demonstrated a significant enhancement in anti-cancer efficacy both *in vitro* and *in vivo*. In conclusion, EVs-mediated cocktail treatment could have potential clinical application for cancer therapy. Although we explored and characterized the synergistic anti-cancer mechanism involving DNA damage, ATP inhibition, and ROS generation, the molecular mechanisms underlying the synergistic anticancer effects, such as apoptosis-related pathways including caspase-3 activation, suppression of anti-apoptotic gene Bcl-2, mitochondrial pathway by stimulating cytochrome *c* release, will be further investigated in our future research.

## Supplementary Material

Supplemental Material
