# Supplementary material for: Amplification of anticancer efficacy by co-delivery of doxorubicin and lonidamine with extracellular vesicles
Source: Drug Deliv. 2022 Jan 5;29(1):192–202. doi: 10.1080/10717544.2021.2023697 (PMC8741238; doi:10.1080/10717544.2021.2023697)
Supplement: Supplemental Material [file IDRD_A_2023697_SM8910.docx]

Supporting Information

**Amplification of anticancer efficacy by co-delivery of**

**doxorubicin and lonidamine with extracellular vesicles**

Huizhen Li^1,5^, Wan Xu^1^, Fang Li^2*^, Ru Zeng^3^, Xiuming Zhang^1^, Xianwu Wang^4^, Shaojun Zhao^4^, Jian Weng^1^, Zhu Li^4*^, Liping Sun^1*^

^1^Department of Biomaterials, The Higher Educational Key Laboratory for Biomedical Engineering of Fujian Province, Research Center of Biomedical Engineering of Xiamen, College of Materials, Xiamen University, Xiamen, China

^2^Key Laboratory of Marine Genetic Resources, Third Institute of Oceanography, Ministry of Natural Resources, Xiamen, China

^3^Department of medical oncology, The First Affiliated Hospital of Xiamen University, Xiamen, China

^4^Xiamen Nuokangde Biological Technology Co., Ltd, Xiamen, China

^5^Department of Radiotherapy, the First Affiliated Hospital of Zhengzhou University, Zhengdong Branch, Zhengzhou, Henan, China

**
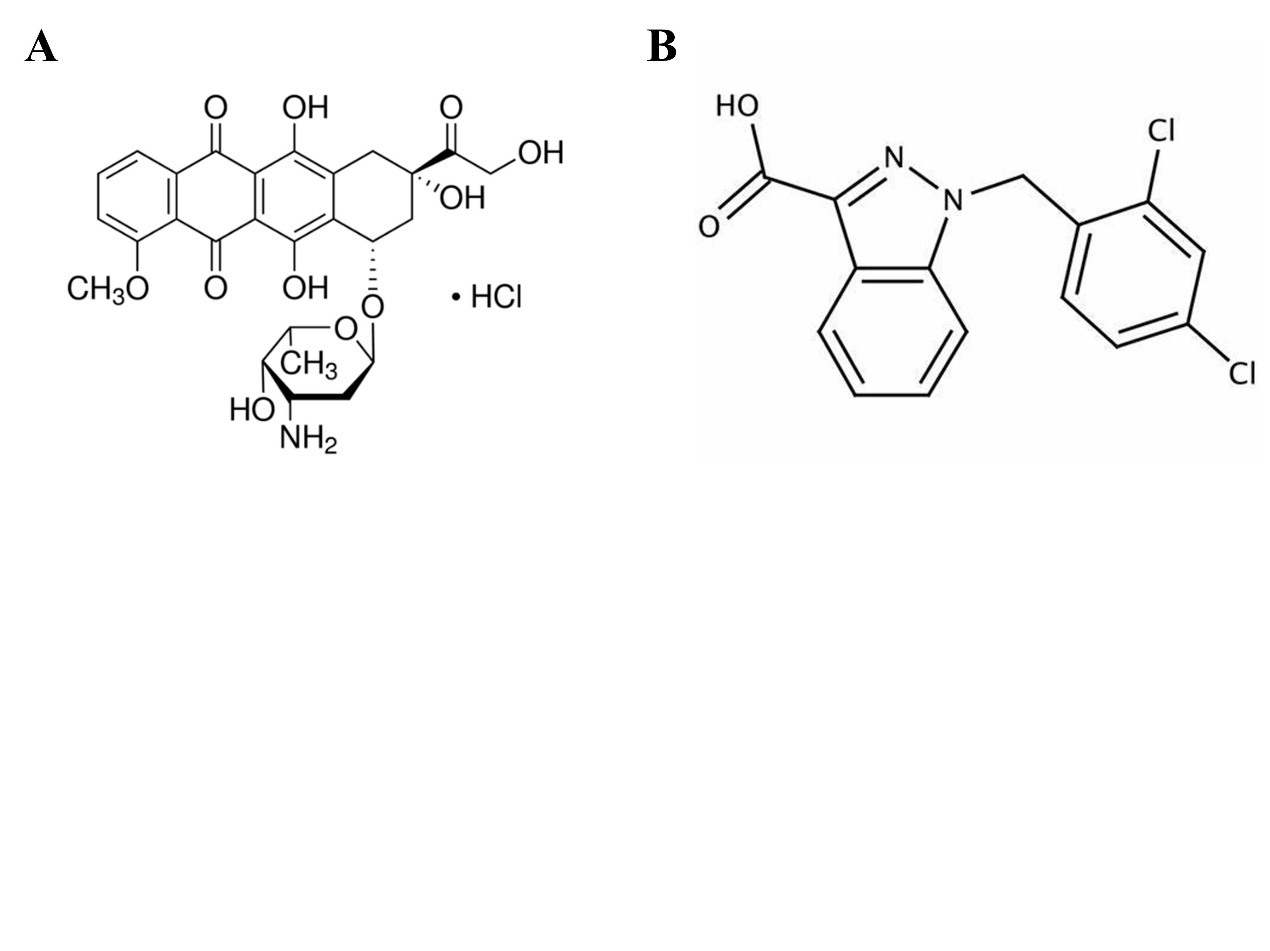
**

**Figure S1.** Molecular structures of (A) DOX，(B) LND.

**
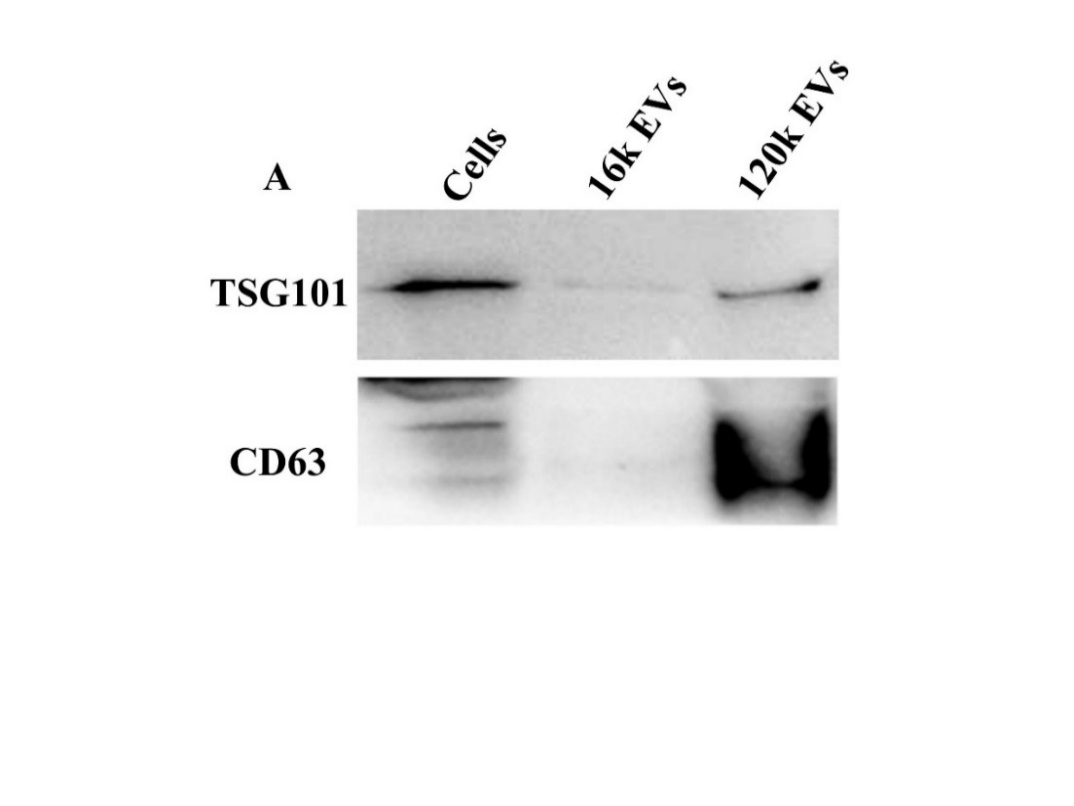
**

**Figure S2.** Characterization of the surface marker of EVs. CD63 and TSG101 expression in A549 whole-cell lysates and EVs were analyzed by western blot.


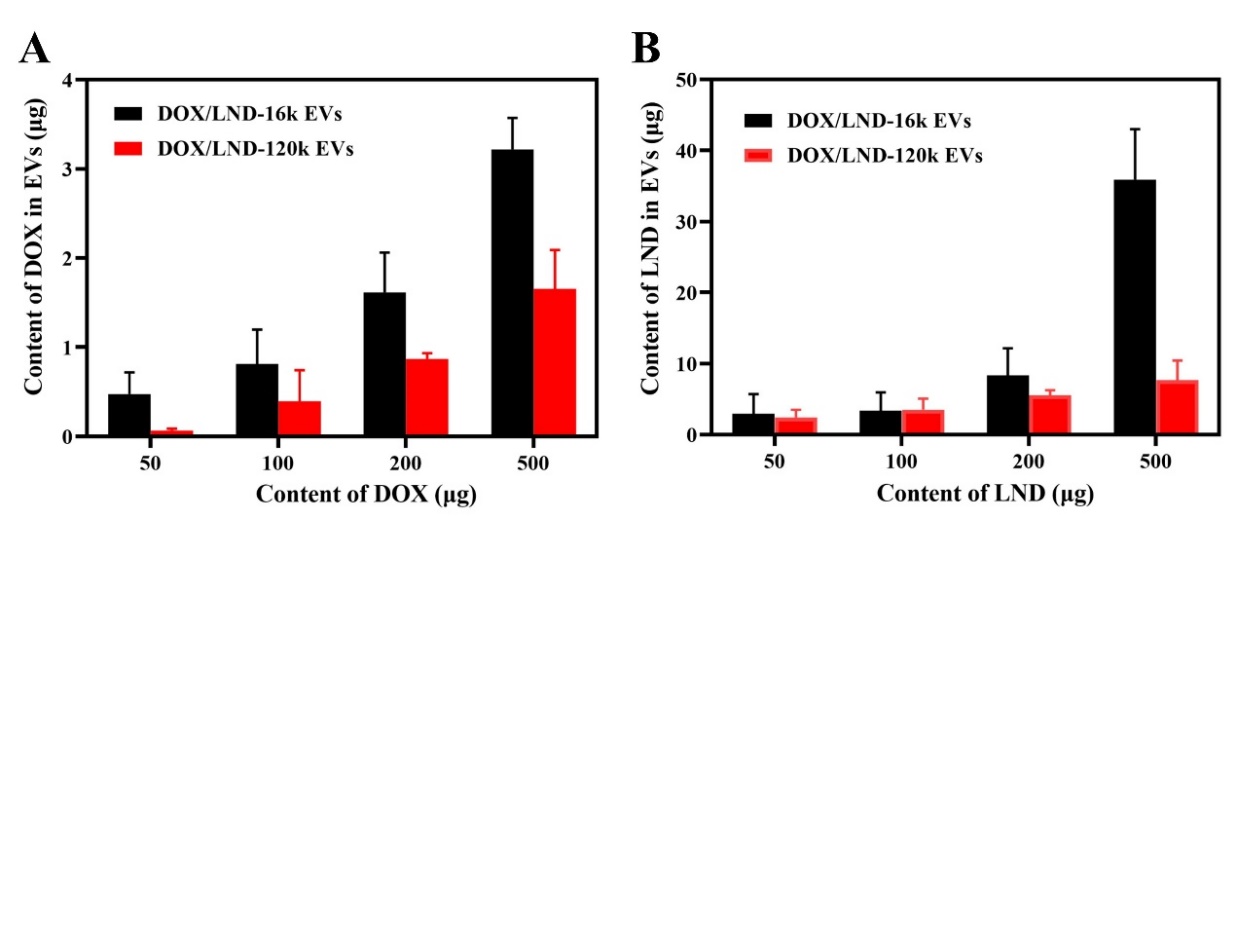


**Figure S3.** (A) The content of DOX loaded in DOX/LND-EVs. (B) The content of LND loaded in DOX/LND-EVs.


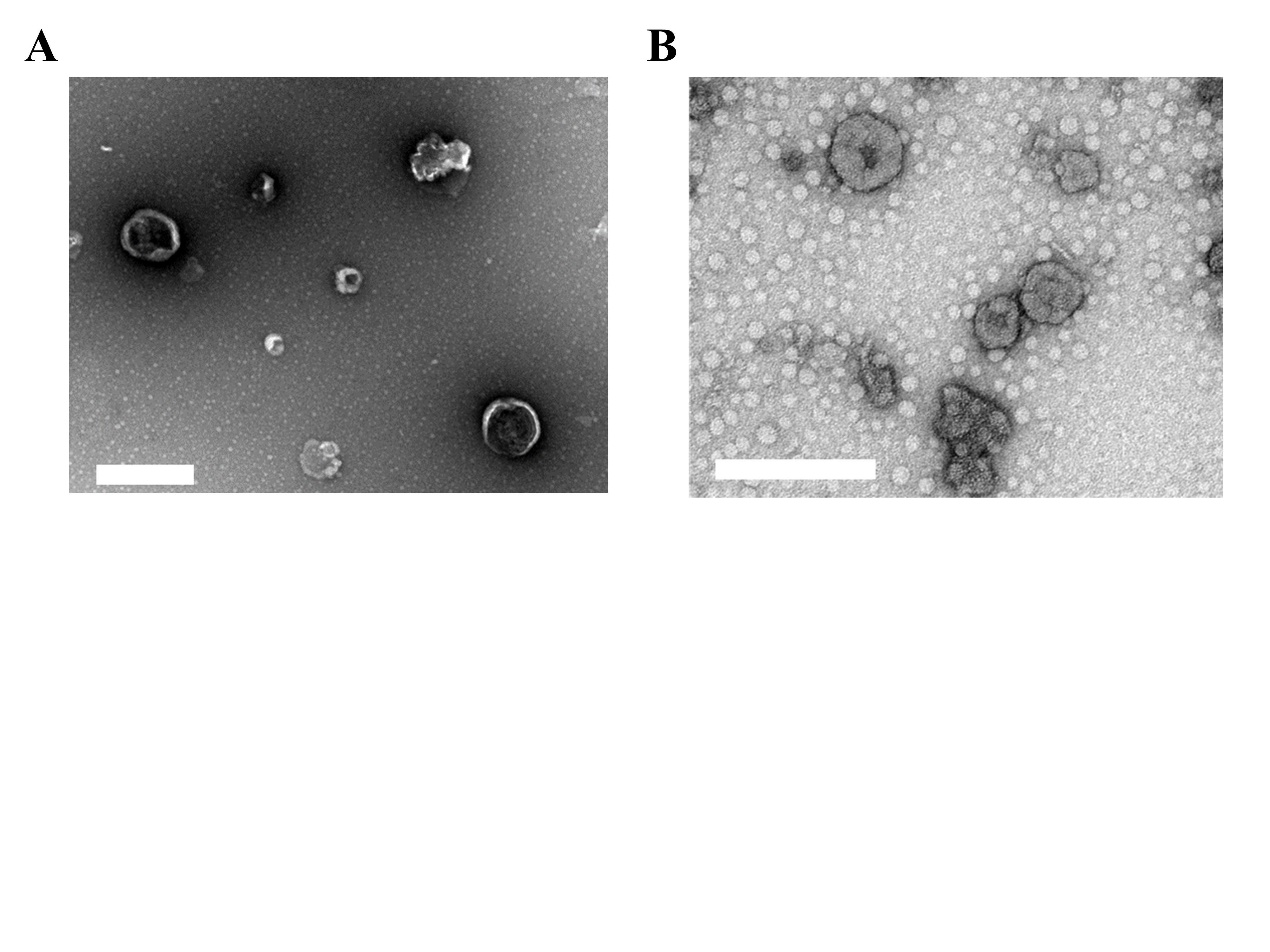


**Figure S4.** TEM image of (A) DOX-16k EVs, (B) DOX-120k EVs. Scale bar: 200 nm.


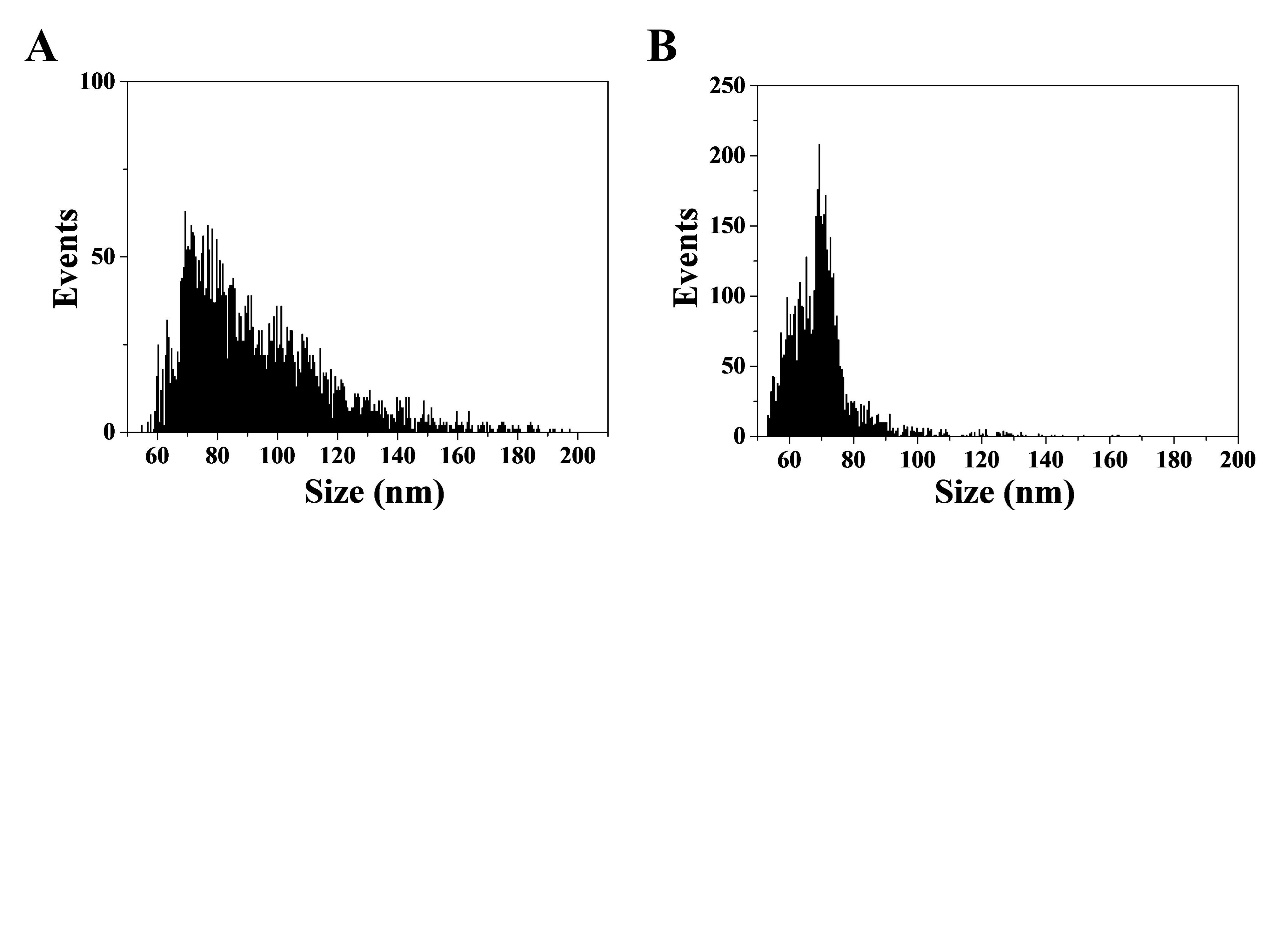


**Figure S5.** Size distribution of (A) DOX/LND-16k EVs, (B) DOX/LND-120k EVs.


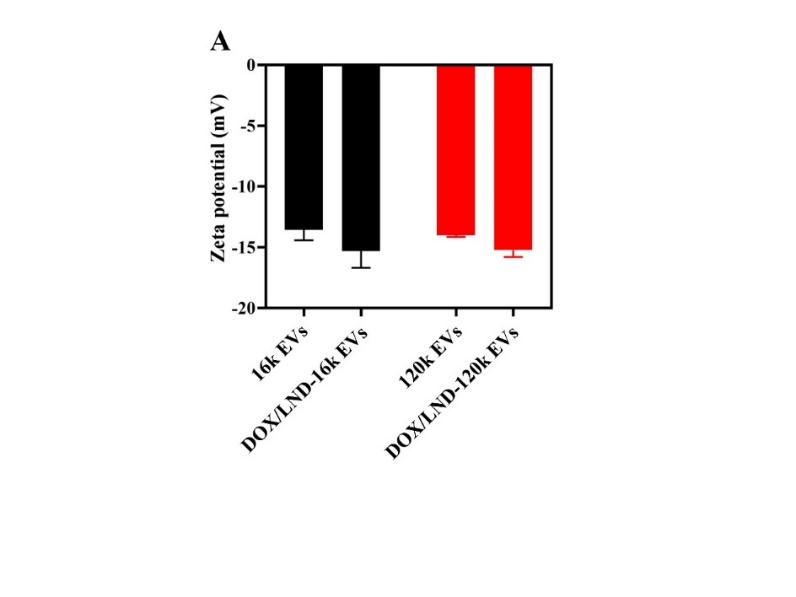


**Figure S6.** Zeta potential of DOX/LND-16k EVs and DOX/LND-120k EVs (n = 3; mean ± SD).


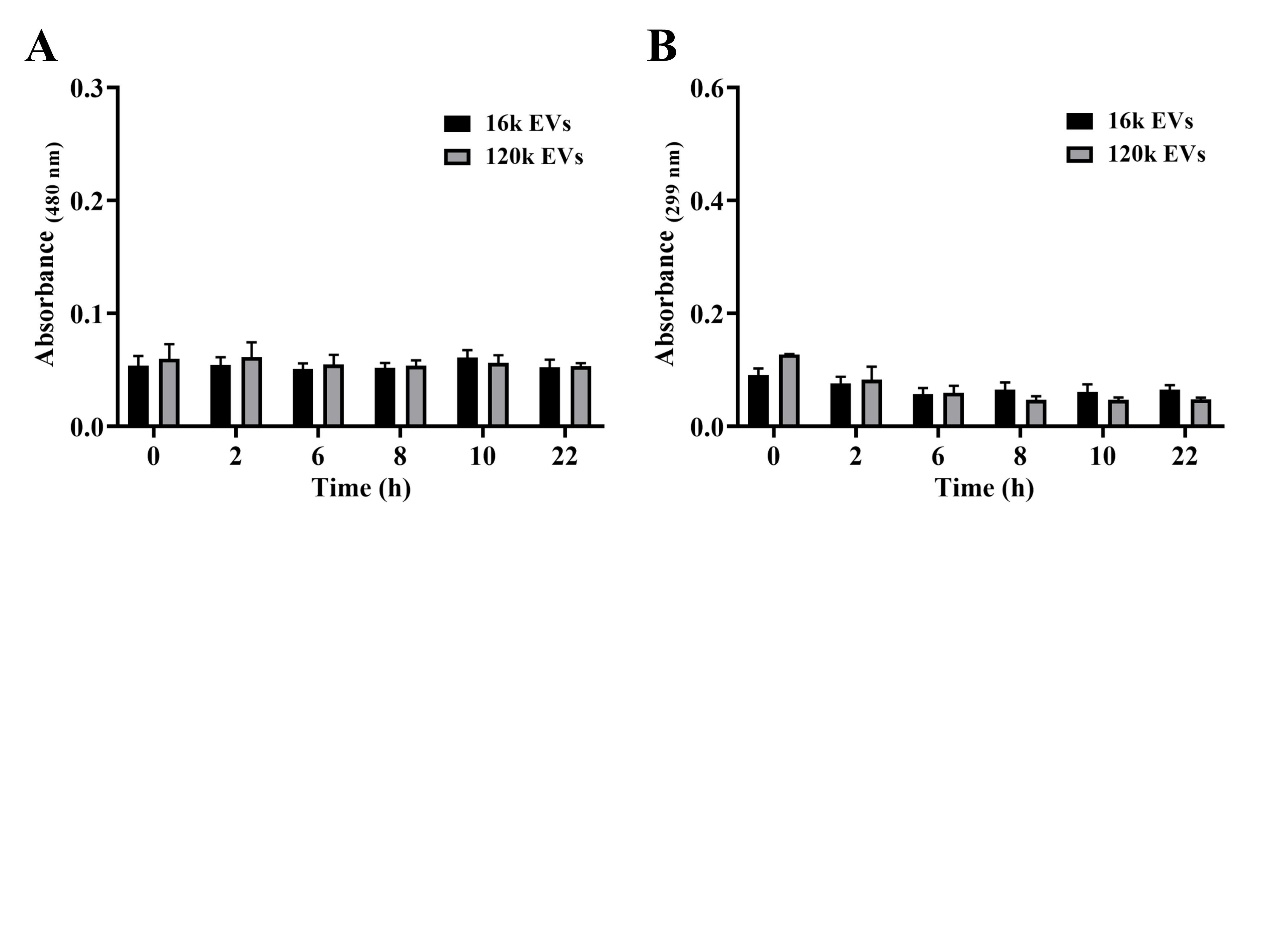


**Figure S7**. (A) The release of DOX loaded in DOX/LND-EVs. (B) The release of LND loaded in DOX/LND-EVs (n = 3; mean ± SD). To test the stability of drug-loaded EVs, the 1.5 mL DOX/LND-EVs solution in PBS was put into a 15 mL Amicon Ultra-15 Centrifugal Filter Unit (Millipore) and then centrifuged at 5000 ×g for 10 min to remove DOX and LND released from EVs at 0h, 2h, 6h,8h, 10h, 22h

**
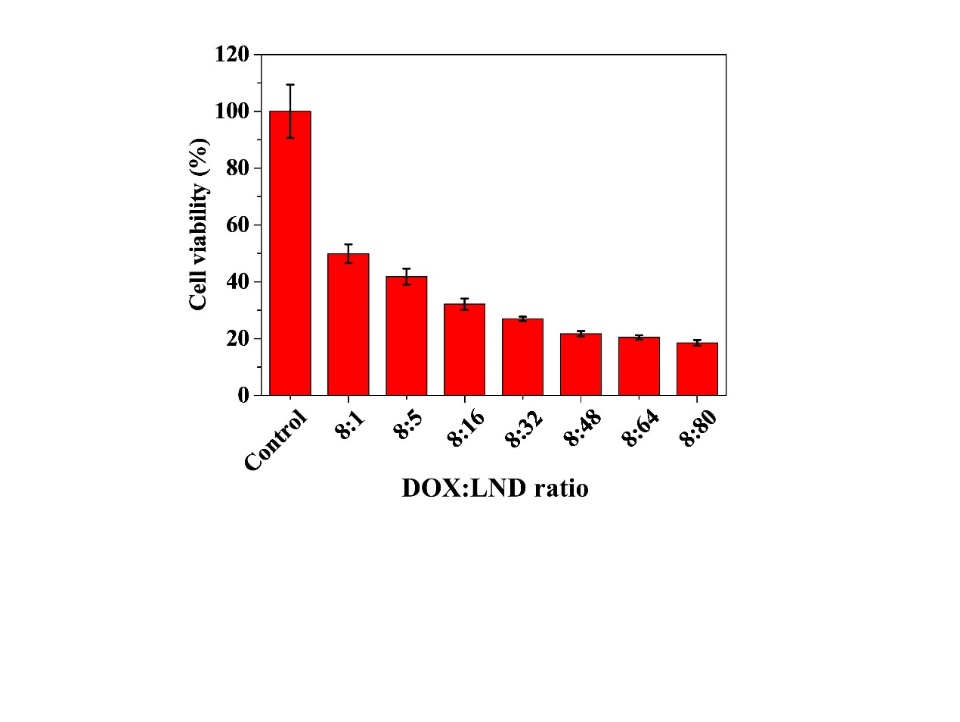
**

**Figure S8.** Optimization of mass ratio of DOX/LND. The DOX concentration is 8 µg/mL, When the DOX/LND ratio arrived to 8:48 (1:6), the cell viability almost reached minimum value (n = 3; mean ± SD).


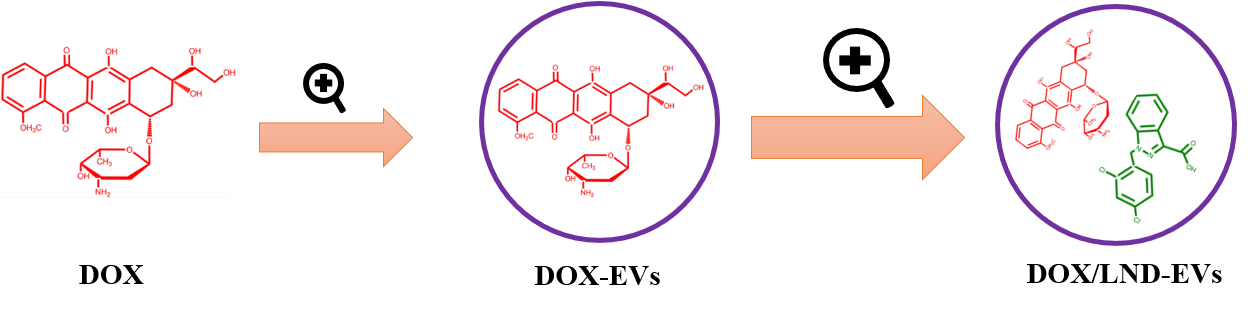
 **Figure S9.** Schematic illustration of the co-delievery of DOX and LND by EVs to amplify the anti-cancer effect. The structural formula of DOX is marked red, while that of LND is marked green. The purple circle indicates the extracellular vesicle.

**Table S1.** Comparison of IC_50_ of free drugs and EVs-encapsulated drugs

| **Sample** | **IC_50_ (μg/mL)** |
| --- | --- |
| DOX  LND | 7.114 (1)  38.758 (0.2) |
| DOX/LND | 2.420 (2.9) |
| DOX-16k EVs | 0.189 (37.7) |
| DOX-120k EVs | 0.262 (27.2) |
| DOX/LND-16k EVs | 0.077 (92.4) |
| DOX/LND-120k EVs | 0.086 (82.7) |

Note: Numbers in the brackets are the relative fold of IC_50_ of DOX compared to other groups.


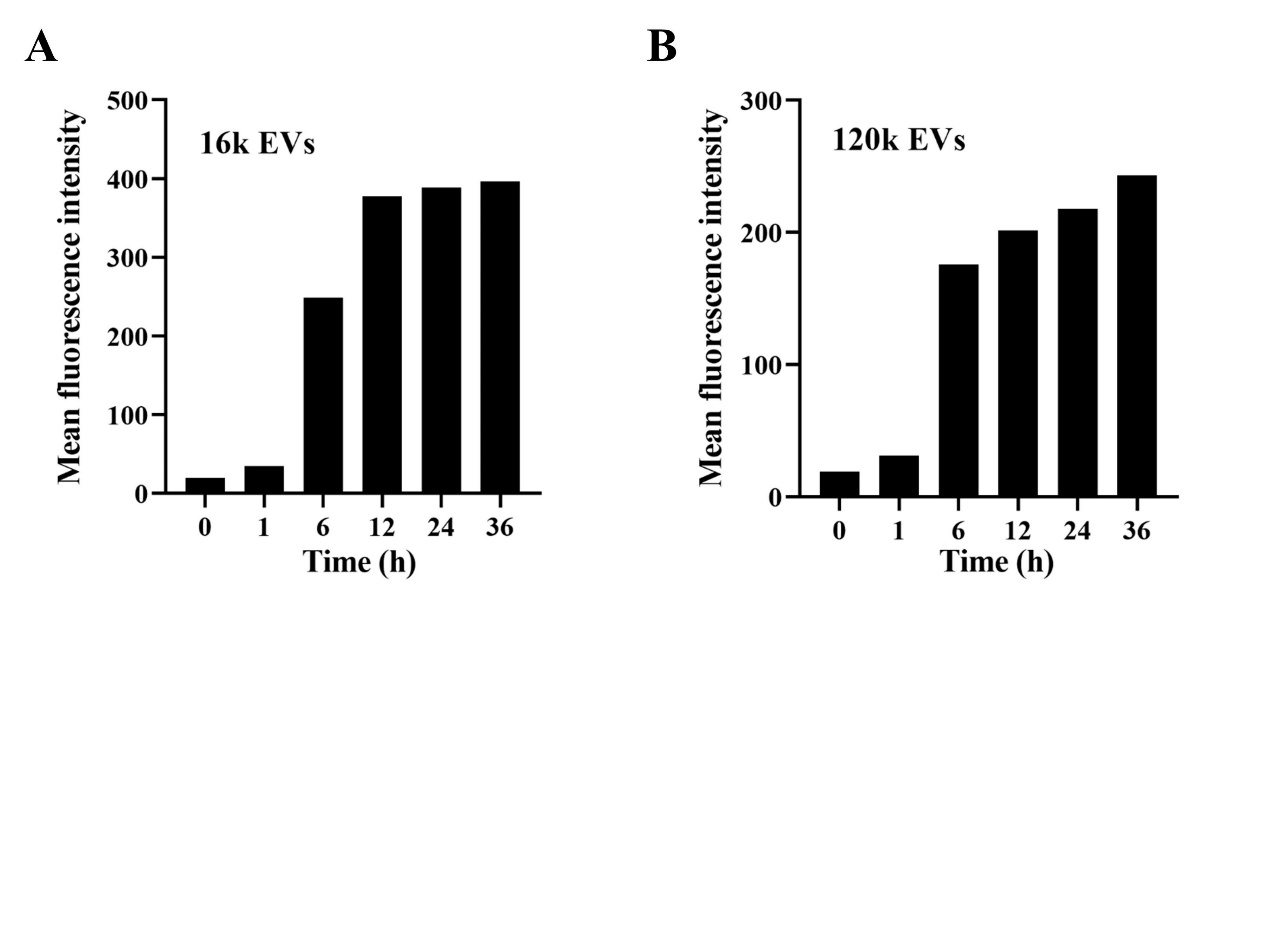


**Figure S10.** Time-dependent internalization of DOX-16K EVs (A) and DOX-120K EVs (B) by A549 cells.

**Table S2.** The Content of cellular drugs uptake

| **SAMPLE** | **Content of drugs in the cell (µg/mL)** | |
| --- | --- | --- |
|  | **DOX** | **LND** |
| DOX/LND | 0.11±0.02（11%） | 2.66±0.28（44%） |
| DOX/LND-16k EVs | 0.84±0.11（84%） | 4.45±0.54（74%） |
| DOX/LND-120k EVs | 0.79±0.08（79%） | 3.61±0.97（60%） |

Note: The concentrations of DOX and LND in the three groups were 1 µg/mL and 6 µg/mL, respectively. The cells were incubated with different different formulations of drugs for 36 h at 37°C. The percentage in the brackets denote the efficiencies of cellular uptake of DOX and LND. (n = 3; mean ± SD).

**Figure S11.** (A) Images of harvested tumor tissues of tumor-bearing mice. (B) Tumor weight of the mice harvested at day 16.  Data were analyzed with non-parametric test and presented as mean ± SD, n = 5 (*p＜0.05, **p＜0.01). (C) H&E staining of major organs and tumor of tumor-bearing mice. Scale bar: 100 µm.


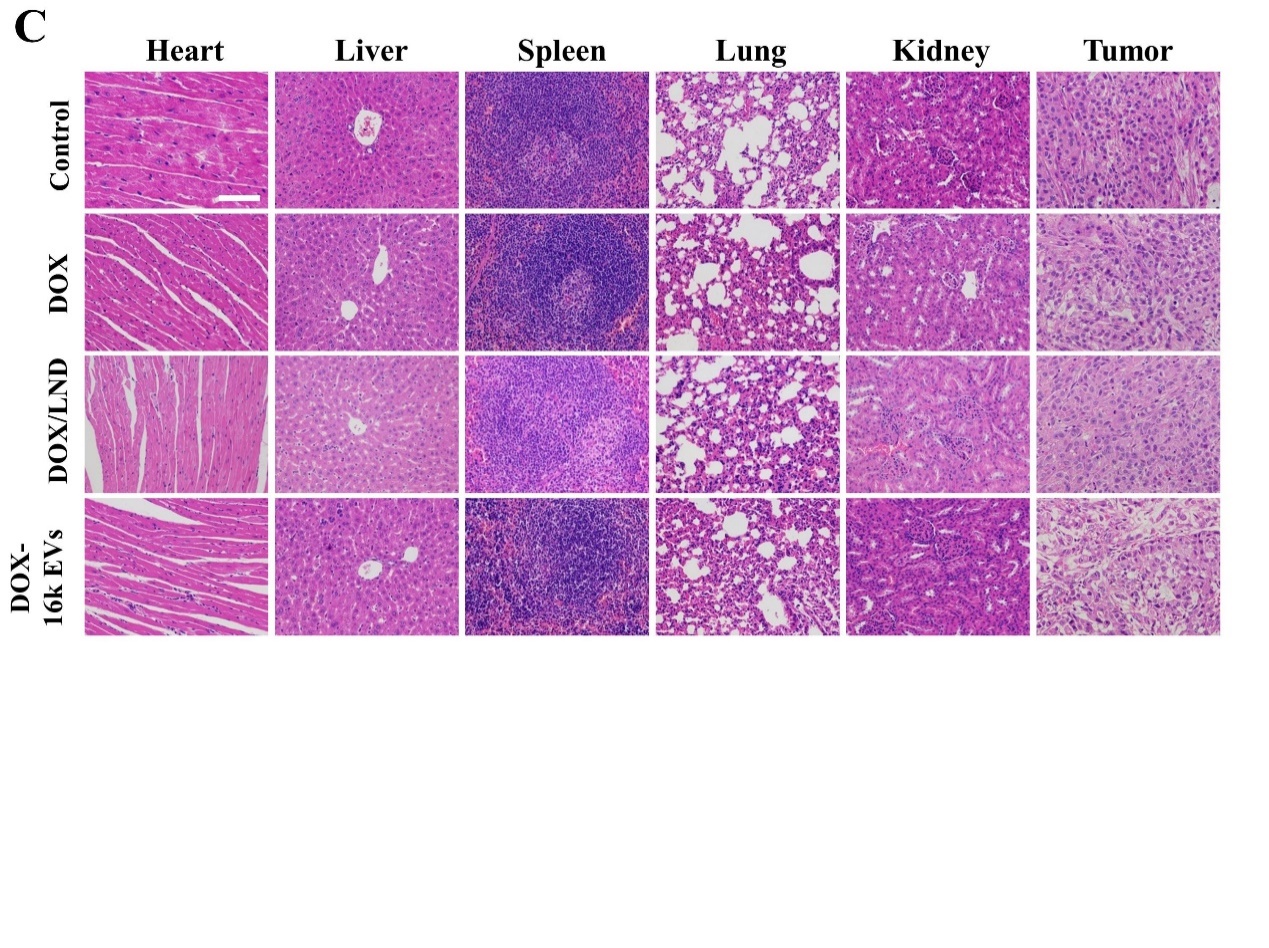

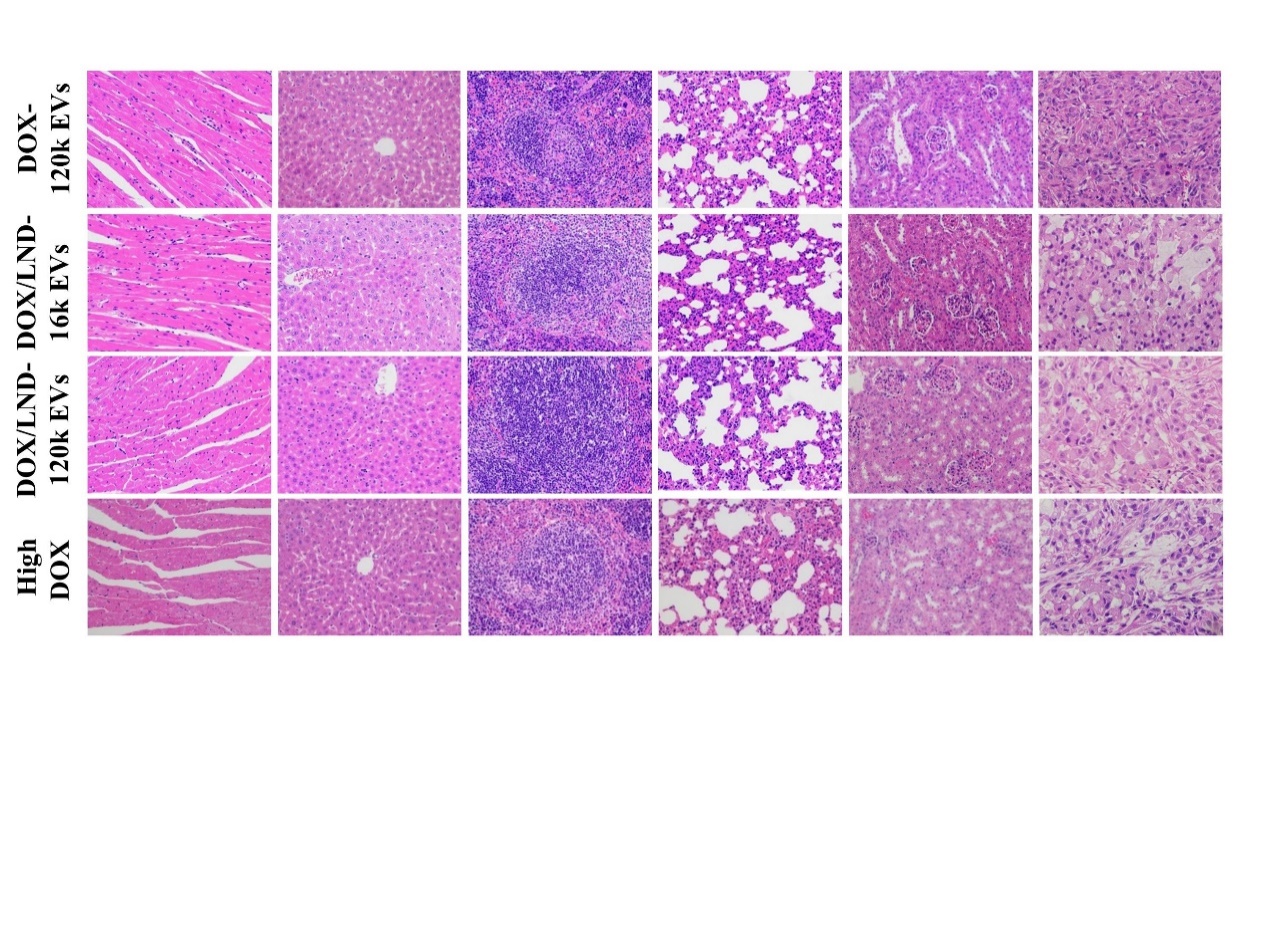

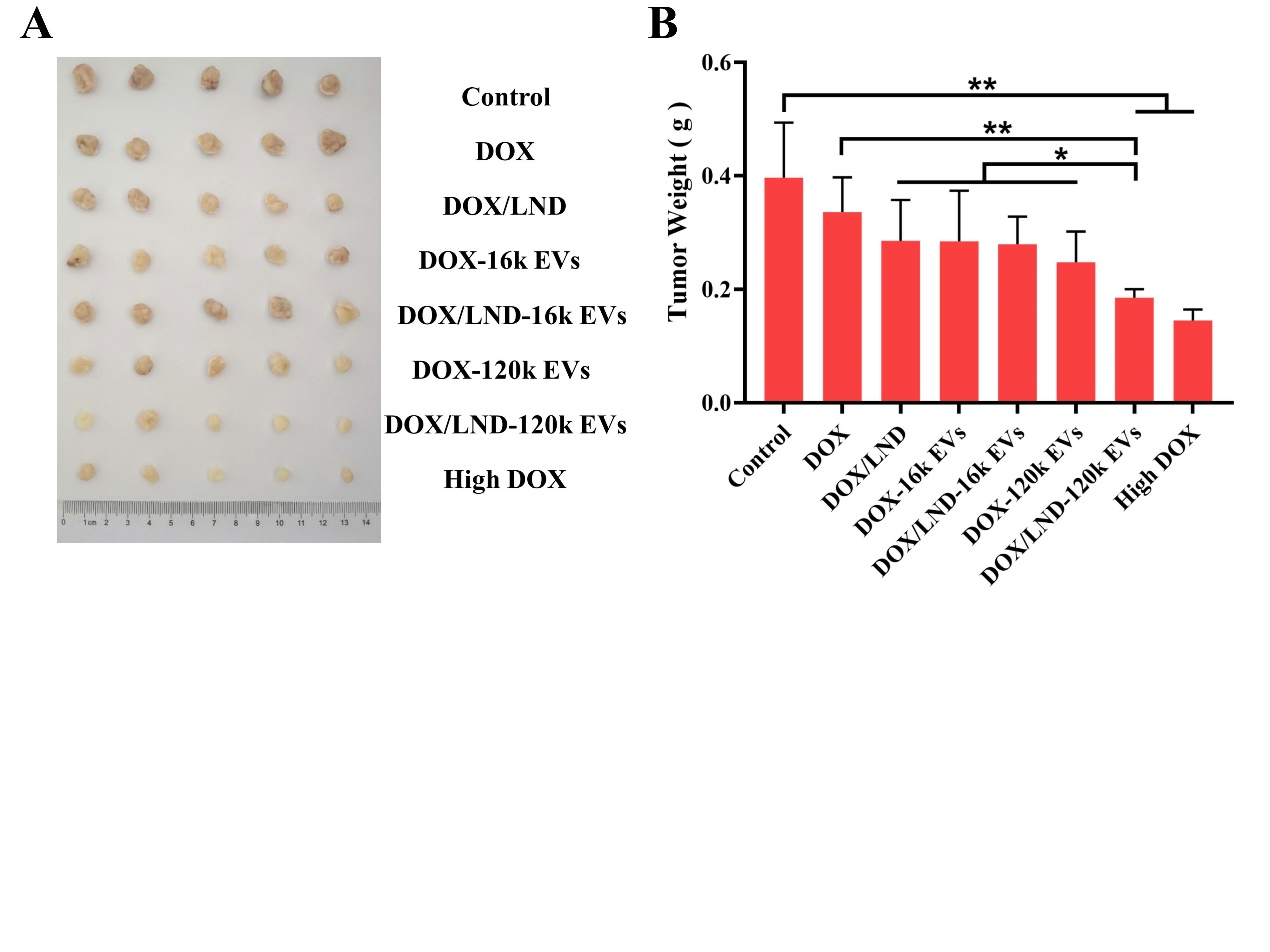


**Table S3.** Relative tumor volume and weight

| **Group** | **Relative tumor volume（%）** | **Relative tumor weight（%）** |
| --- | --- | --- |
| Control | 100±6 | 100±25 |
| DOX (0.1 mg/kg) | 94.71±9 | 85.75±15 |
| DOX/LND | 66.20±4 | 71.96±18 |
| DOX-16k EVs | 66.27±6 | 71.73±22 |
| DOX-120k EVs | 58.42±4 | 62.46±12 |
| DOX/LND-16k EVs | 64.95±5 | 70.40±14 |
| DOX/LND-120k EVs | 53.00±8 | 46.75±4 |
| High DOX (4 mg/kg) | 26.63±2 | 36.60±5 |
